# Binding Sites of miR-1273 Family on the mRNA of Target Genes

**DOI:** 10.1155/2014/620530

**Published:** 2014-08-26

**Authors:** Anatoly Ivashchenko, Olga Berillo, Anna Pyrkova, Raigul Niyazova

**Affiliations:** National Nanotechnology Laboratory, Al-Farabi Kazakh National University, Al-Farabi 71, Almaty 050038, Kazakhstan

## Abstract

This study examined binding sites of 2,578 miRNAs in the mRNAs of 12,175 human genes using the MirTarget program. It found that the miRNAs of miR-1273 family have between 33 and 1,074 mRNA target genes, with a free hybridization energy of 90% or more of its maximum value. The miR-1273 family consists of miR-1273a, miR-1273c, miR-1273d, miR-1273e, miR-1273f, miR-1273g-3p, miR-1273g-5p, miR-1273h-3p, and miR-1273h-5p. Unique miRNAs (miR-1273e, miR-1273f, and miR-1273g-3p) have more than 400 target genes. We established 99 mRNA nucleotide sequences that contain arranged binding sites for the miR-1273 family. High conservation of each miRNA binding site in the mRNA of the target genes was found. The arranged binding sites of the miR-1273 family are located in the 5′UTR, CDS, or 3′UTR of many mRNAs. Five repeating sites containing some of the miR-1273 family's binding sites were found in the 3′UTR of several target genes. The oligonucleotide sequences of miR-1273 binding sites located in CDSs code for homologous amino acid sequences in the proteins of target genes. The biological role of unique miRNAs was also discussed.

## 1. Introduction

Once a microRNA (miRNA) has been discovered, the number of publications devoted to clarifying its biological role increases constantly and quickly [1]. Researchers are interested in miRNAs because they participate in the posttranscription regulation of gene expression [2]. These nanoscale molecules participate, directly or indirectly, in almost all key organism processes [1–3]. Identifying the target genes of a miRNA is an imperfect process, and some programs predict a large number of false-positive binding sites. Additionally, some papers have discussed the existence of miRNA binding sites only in the 3′-untranslated region (3′UTR) and the obligatory presence of a “seed” in the 5′ end of the miRNA, but these statements and others are poorly substantiated [4, 5]. The binding sites located in coding domain sequences (CDSs) of mRNAs appeared recently [6]. The process of establishing a miRNA's precise biological function is slow because they are poorly understood, despite the large number of publications devoted to them. Because miRNAs regulate gene expression, they participate in many pathological processes [7–17]. Changes in the miRNA concentration have been shown to occur during the development of breast [7], lung [8], esophageal [9], stomach [10], intestine [11], prostate [12], and other cancers [13–15]. Changes in the interactions between the miRNAs and mRNAs of oncogenes [16] and genes suppressors [17] have been shown to cause malignant diseases. Thus, it is necessary to clarify the role of miRNAs in disease development.

In this work, we studied the binding of 2,578 miRNAs with 12,175 mRNAs for genes. The majority of these genes participate in the development of lung cancer, breast cancer, gastrointestinal cancer, and others. First, it is necessary to determine the features of miRNA binding sites. One miRNA can bind to one or more mRNAs, and some mRNAs have multiple binding sites for different miRNAs that are within the same family. The expression of most human protein-coding genes depends directly or indirectly on more than 2,500 miRNAs. We must also establish whether the connections between the miRNAs and mRNAs are minor and only affect individual genes or whether they are organized to regulate system-wide gene expression. Specifically, the relationships between the binding sites of one family of miRNAs and all of the mRNA sites must be elucidated.

## 2. Materials and Methods

Human miRNAs (hsa-miRNAs) were taken from the miRBase site (http://mirbase.org). The mRNAs for human genes were taken from the GenBank database (http://www.ncbi.nlm.nih.gov) using Lextractor002 script (http://sites.google.com/site/malaheenee/software). The target genes for the tested miRNAs were revealed using the MirTarget program, which was developed in our laboratory. This program defines the following features of binding: (a) the beginning of a miRNA binding with mRNAs; (b) the localization of miRNA binding sites in the 5′-untranslated regions (5′UTRs), CDSs and 3′UTRs of the mRNAs; (c) the free energy of hybridization (Δ*G*, kJ/mole); and (d) the schemes of nucleotide interactions between the miRNAs and the mRNAs. The ratio Δ*G*/Δ*G*_*m*_ (%) was counted for each site, where Δ*G*_*m*_ equaled the free energy of a miRNA binding with its perfect complementary nucleotide sequence. The miRNA binding sites located on the mRNAs had Δ*G*/Δ*G*_*m*_ ratios of 90% and more. We note the position of the binding sites on the mRNA, beginning from the first nucleotide of the mRNA's 5′UTR. It found bonds between adenine (A) and uracil (U), guanine (G) and cytosine (C), and G and U, as well as between A and C via a hydrogen bond [18]. The distance between A and C was equal to the G-C, A-U, and G-U distances [19]. The numbers of hydrogen bonds in the G-C, A-U, G-U, and A-C interactions were taken to be 3, 2, 1, and 1, respectively. The free binding energies of these nucleotide pairs were accepted as the same values (3 : 2 : 1 : 1).

## 3. Results and Discussion

### 3.1. Features of the miR-1273 Family

The binding powers between the 2,578 tested hsa-miRNAs and the mRNAs from 12,175 human genes were calculated. Some members of the miR-1273 family have a greater number of target genes than others. For example, miR-1273g-3p and miR-1273f can bind to 1,074 and 766 genes, respectively, with Δ*G*/Δ*G*_*m*_ ratios of 90% and more. Other miRNAs have some target genes. For example, 1271-5p and 1271-3p have only six and nine target genes, respectively. The miRNAs with over 400 target genes were called unique miRNAs (umiRNAs). In addition, the binding sites for these unique miRNAs are unusually located in the mRNAs. Members of the miR-1273 family have different origins, lengths, quantities, and properties of the miRNA binding sites, among other features. Some characteristics of the miR-1273 family are outlined below.

With a length of 25 nt, miR-1273a is coded in an intron of the regulator of G-protein signaling 22 gene (*RGS22*), located on chromosome 8. We found that miR-1273a has 154 binding sites on 148 target mRNAs; thus, some of the mRNAs have two binding sites. Of those, 146 miR-1273a binding sites are located in 3′UTRs, six sites are located in 5′UTRs, and two sites are located in CDSs.

With a length of 22 nt, miR-1273c is coded in an intron of the T cell lymphoma invasion and metastasis 2 gene (*TIAM2*), located on chromosome 6. We found that 84 target gene mRNAs have one binding site for miR-1273c, while* GOLGA3* has 2 sites, for a total of 86 miR-1273c sites. Seven of those are located in 5′UTRs, two sites are located in CDSs, and 76 sites are located in 3′UTRs.

With a length of 25 nt, miR-1273d is coded in an intron of the Kinesin family member 1B gene (*KIF1B*), located on chromosome 1. We found that 114 target gene mRNAs have one binding site, while* ARGFX* mRNA has two sites, for a total of 116 miR-1273d sites. Six of those are located in 5′UTRs, five sites are located in CDSs, and 104 sites are located in 3′UTRs.

With a length of 22 nt, miR-1273e's origin was not established. We found 449 miR-1273e binding sites on the mRNAs of 413 target genes. Of those, 19 binding sites are located in 5′UTRs, nine sites are located in CDSs, and 421 sites are located in 3′UTRs.

With a length of 19 nt, miR-1273f is coded in an intron of the sterol carrier protein 2 (*SCP2*) gene, located on chromosome 1. We found that the mRNAs of 766 genes contain 886 miR-1273f binding sites. Of those, 45 sites are located in 5′UTRs, 40 sites are located in CDSs, and 801 sites are located in 3′UTRs. The mRNAs of ten genes have completely complementary binding sites for miR-1273f. Each mRNA of the* GNL3L, IRGQ, ORAI2, *and* PLCXD1 *genes has four miR-1273f binding sites that are located in 3′UTRs.

With a length of 21 nt, miR-1273g-3p is coded in an intron of the* SCP2* gene, located on chromosome 1. We found that miR-1273g-3p has 1,330 binding sites on 1,074 mRNAs. Of those, 69 miR-1273g-3p binding sites are located in 5′UTRs, 38 sites are located in CDSs, and 1,223 sites are located in 3′UTRs. The mRNAs of seven genes have completely complementary binding sites for miR-1273g-3p. The mRNAs of the* NOL9, PLCXD1, ZNF490, CYP20A1, GNL3L, PPM1K, RBMS2, SAR1B, *and* SLC35E2* genes have four binding sites. The* IRCQ* and* ZNF850* genes have five binding sites, and the mRNA of the* MDM4* gene has six miR-1273g-3p binding sites. All of these sites are located in 3′UTRs.

With a length of 22 nt, miR-1273g-5p is coded in an intron of the* SCP2* gene, located on chromosome 1. The mRNAs of 33 target genes have one miR-1273g-5p binding site. Two of those sites are located in 5′UTRs, five sites are located in CDSs, and 26 sites are located in 3′UTRs.

With a length of 21 nt, miR-1273h-3p is coded in the intergenic nucleotide sequence of chromosome 16. We found that miR-1273h-3p has 38 target genes. The mRNA of these target genes have only one miR-1273h-3p binding site. Three sites are located in 5′UTRs and 35 sites are located in 3′UTRs, but no sites were found in CDSs.

With a length of 21 nt, miR-1273h-5p is coded in the intergenic sequence of chromosome 16. We found that miR-1273h-5p has 127 binding sites on 126 target gene mRNAs. Eleven sites are located in 5′UTRs, 14 sites are located in CDSs, and 102 sites are located in 3′UTRs.

### 3.2. Arrangement of the miR-1273 Family's Binding Sites in the mRNA of Target Genes

This study revealed that several hundred mRNAs have homologous nucleotide sequences containing binding sites for members of the miR-1273 family. Two miRNA binding sites located on one mRNA were termed pair sites. Specifically, we examined pairs composed of miR-1273g-3p with another member of the miR-1273 family. Data about the localization of these pair sites are presented in the text below. These arranged pair sites are located in mRNA segments that have a length of just 99 nucleotides.

**Figure 1:**
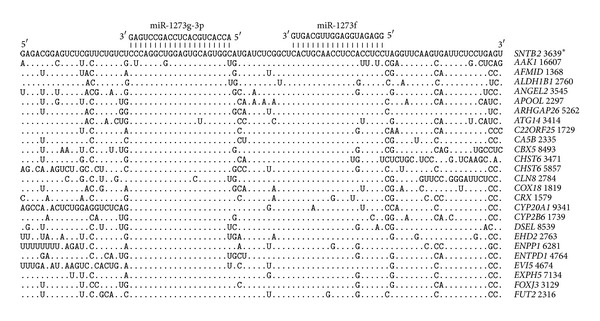
Arranged binding sites miR-1273g-3p and miR-1273f in 3′UTR mRNA target genes. Note Figures 1–11. Symbol | is hydrogen bonds between nucleotides miRNA and mRNA;  * is position of binding sites miR-1273g-3p on mRNA; (.) equals nucleotide.

The mRNAs of 582 general target genes have pair sites for both miR-1273g-3p and miR-1273f. Of those, 24 mRNAs are located in 5′UTRs, 18 are located in CDSs, and 540 are located in 3′UTRs. The locations of the miR-1273g-3p and miR-1273f binding sites in the 3′UTRs of mRNAs are presented in Figure 1. The nucleotide sequence in the 3′UTR of the* SNTB2 *gene that contained this pair binding site is chosen for comparison with the pair sites of other mRNAs. Most binding sites have nucleotide replacements (purine to purine and pyrimidine to pyrimidine) to retain their hydrogen bonds. Figure 1 shows that the miR-1273g-3p and miR-1273f binding sites in all of the tested mRNAs are located at distance of 12 nucleotides. The nucleotide sequences of these revealed that pair sites are highly homologous, indicating that their origins are not casual.

**Figure 2:**
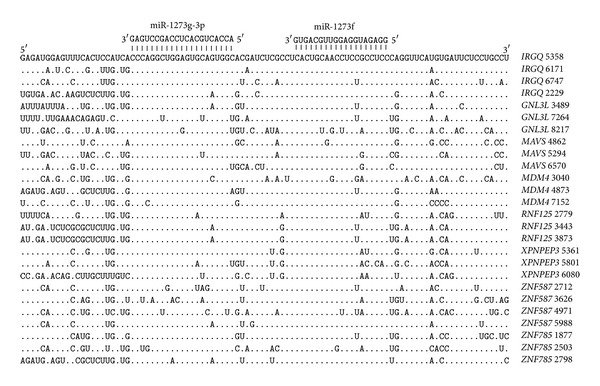
Arranged binding sites miR-1273g-3p and miR-1273f in 3′UTR mRNA that contain three and four pair binding sites.

The mRNAs of many genes contain two or more pair sites for miR-1273g-3p and miR-1273f. The nucleotide sequences of sites in mRNA 3′UTRs that contain three and four arranged pairs of sites for these two miRNAs are shown in Figure 2. The 3′UTR of the* IRGQ *gene, for example, has six pair sites. The nucleotide sequences of the repeating pair binding sites have a high degree of homology, again testifying that the origin of these sites in the 3′UTR is not random. The distance between the binding sites is still 12 nucleotides.

**Figure 3:**
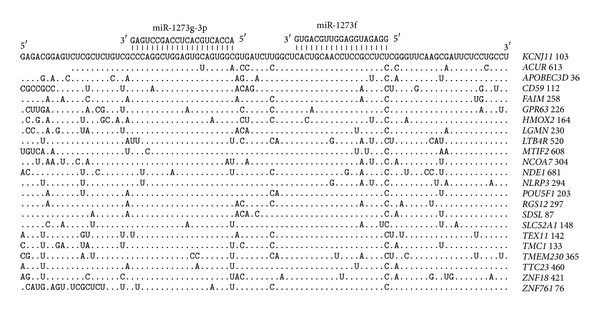
Arranged binding sites miR-1273g-3p and miR-1273f in 5′UTR mRNA target genes.

The 5′UTRs of 24 genes also have pair binding sites for miR-1273g-3p and miR-1273f (Figure 3). The nucleotide sequences of the sites in the 5′UTRs also have a high degree of homology. The distance between the binding sites is 12 nucleotides, indicating that both the 5′UTR and 3′UTR binding sites have a common origin.

**Figure 4:**
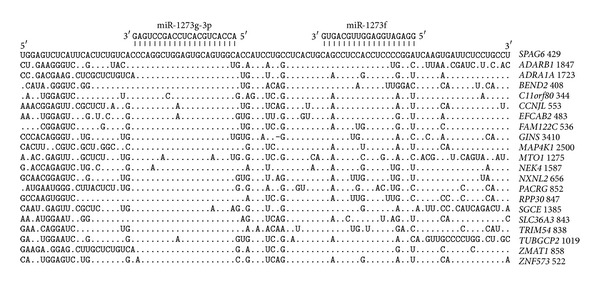
Arranged binding sites miR-1273g-3p and miR-1273f in CDS mRNA target genes.

The miR-1273g-3p and miR-1273f pair binding sites are present in the CDSs of 12 genes, and their locations are presented in Figure 4. The distance between the binding sites is again 12 nucleotides. The nucleotides of the miR-1273g-3p and miR-1273f binding sites in CDSs are less homologous than those located in the 5′UTRs and 3′UTRs. However, it is still possible to suppose a general origin for all of the pair sites located in the CDSs, 5′UTRs, and 3′UTRs.

**Figure 5:**
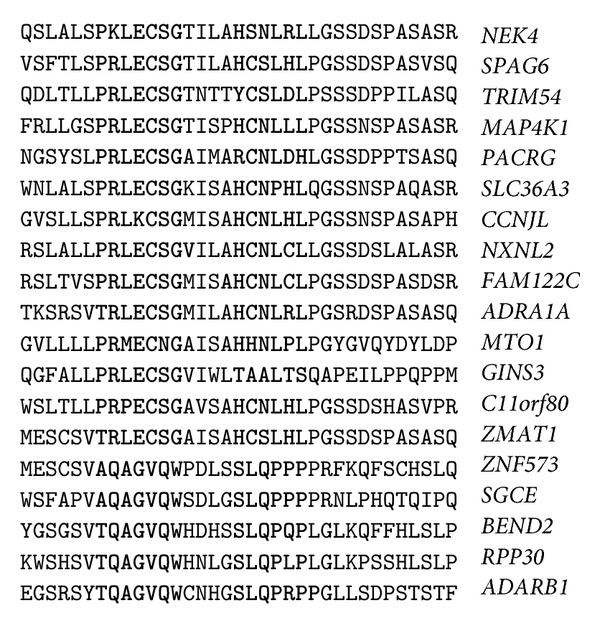
Amino acid sequences are coded by the segment of mRNA that corresponds to miR-1273g-3p and miR-1273f binding sites.

The nucleotide sequences of the binding sites in CDSs are translated into corresponding amino acid sequences that create proteins. If the nucleotides of the miR-1273g-3p binding sites are read in different open reading frames (ORFs), three different oligopeptides can be produced. The oligonucleotide 5′-CUCAGGCUGGAGUGCAGUGGU-3′ of miR-1273g-3p's binding site can code the LRLECSG, SGWSAVV, and QAGVQW oligopeptides. The mRNAs of 14 genes have ORF oligopeptides that are homologous to RLECSG (Figure 5). Six mRNAs have other ORF and code oligopeptides that are homologous to QAGVQW. The third ORF was found only in the* NOP2* gene. The amino-acid sequences adjoining the studied oligopeptides are also homologous in some proteins. For example, in the ZNF573 and ZMAT1 proteins, the MESCSV hexapeptide is located near the TRLECSG and AQAGVQW oligopeptides, which corresponds to the nucleotides of the miR-1273g-3p binding sites. The oligonucleotide 5′-CACUGCAACCUCCAUCUCC-3′, in the miR-1273f binding site, can code the HCNLHL, TATSIS, and SLQPPS oligopeptides. In 5 genes that contain the miR-1273f binding site in their CDSs, the oligonucleotides code homologous oligopeptides in all three ORFs (Figure 5).

The homology of the nucleotide sequences adjacent to the miR-1273f binding sites causes the homology of the corresponding oligopeptides. The mRNA part between the miR-1273g-3p and miR-1273f binding sites codes homologous tripeptides (DLG and ILA) and tetrapeptides (AISA in both the MTO1 and ZMAT1 proteins). The nucleotide sequences of the mRNA segments adjacent to the miR-1273f site code homologous oligopeptides in some proteins. For example, the PGSSDS hexapeptide is located in both the ZMAT1 and C11orf80 proteins, the GSSNSPA heptapeptide is located in the MAP4K1 and SLC36A3 proteins, and the GSSDSPAS nonapeptide is located in the NEK4, SPAG6, FAM122C, and ZMAT1 proteins.

**Figure 6:**
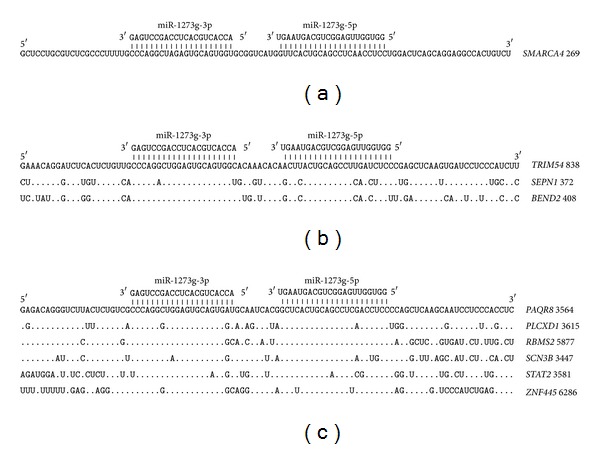
Arranged binding sites miR-1273g-3p and miR-1273g-5p in 5′UTR (a), CDS (b), and 3′UTR (c) mRNA target genes.

The 3′UTR of 16 genes have pair binding sites for miR-1273g-3p and miR-1273g-5p. The mRNA of the* PAQR8* gene is chosen to compare with sites from other mRNAs (Figure 6). This mRNA can form hydrogen bonds with all of the nucleotides in both the miR-1273g-3p and miR-1273g-5p binding sites. The miR-1273g-3p and miR-1273g-5p binding sites in the 3′UTR have a high degree of homology. The distance between the binding sites is 9 nucleotides, indicating a general origin of these pair binding sites in the 3′UTR of the studied genes. The 5′UTR of* SMARCA4* has paired miR-1273g-3p and miR-1273g-5p binding sites (Figure 6). All of the nucleotides in the binding sites of these miRNAs form hydrogen bonds. The CDSs of 4 genes have paired miR-1273g-3p and miR-1273g-5p binding sites (Figure 6). Homologous oligonucleotides in the miR-1273g-3p binding sites coded the homologous oligopeptides PRLECSG and QAGVQW through two ORFs (Figure 6).

**Figure 7:**
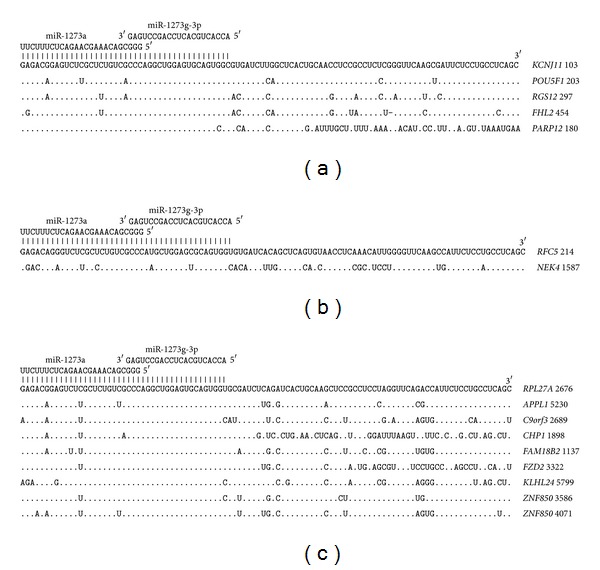
Arranged binding sites miR-1273g-3p and miR-1273a in 5′UTR (a), CDS (b), and 3′UTR (c) mRNA target genes.

Both miR-1273g-3p and miR-1273a have pair binding sites in the mRNA of 113 genes. Five pair binding sites are located in mRNA 5′UTRs (Figure 7). The nucleotide sequences of these binding sites have three common nucleotides that are identical in five mRNAs. A high degree of homology was found in 99 nucleotide segments of the 5′UTR of the* KCNJ11, POU5F1, RGS12,* and* FHL2* genes. Only half of the binding sites located in the 5′UTRs of the* PARP12* gene are highly homologous. The CDSs of two genes contain pair binding sites for miR-1273g-3p and miR-1273a (Figure 7). Both of these gene sites are highly homologous and have three overlapped nucleotides. These sites can also code homologous polypeptides.

**Figure 8:**
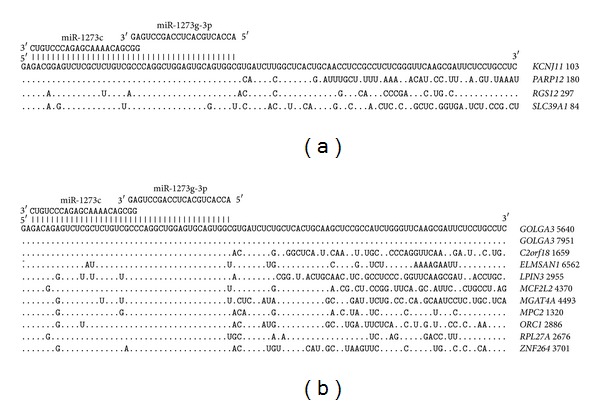
Arranged binding sites miR-1273g-3p and miR-1273c in 5′UTR (a) and 3′UTR (b) mRNA target genes.

The 3′UTR of target genes have paired miR-1273g-3p and miR-1273a binding sites that are also located in the 5′UTR, with three overlapped nucleotides. The miR-1273g-3p and miR-1273a sites in the 3′UTR are highly homologous. The 3′-end sites also have homology with the nucleotides in the mRNA of many genes. The mRNAs of four genes have paired miR-1273g-3p and miR-1273c binding sites located in their 5′UTRs; two nucleotides are common to two sites (Figure 8). The nucleotide sequences of the binding sites are identical in the target genes' mRNAs. Other portions of the mRNA also have homologous nucleotide sequences. The location of paired miR-1273g-3p and miR-1273c binding sites is identical in both the mRNA 5′UTRs and the 3′UTRs (Figure 8). The homology of the nucleotide sequences in the binding sites is high. The nucleotide sequences adjacent to the miR-1273g-3p binding site are also very homologous.

**Figure 9:**
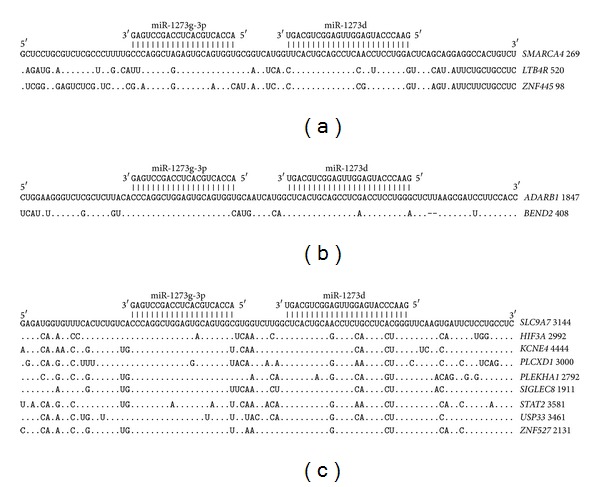
Arranged binding sites miR-1273g-3p and miR-1273d in 5′UTR (a), CDS (b), and 3′UTR (c) mRNA target genes.

The paired miR-1273g-3p and miR-1273d binding sites are located in the 5′UTR at a distance of 13 nucleotides (Figure 9). The homology of the nucleotide sequences in the binding sites is high. The segments of mRNA at the 5′-end of the miR-1273d binding site, consisting of 10 nucleotides to one side and 18 nucleotides to the other, have only three different nucleotides. We assume that there is a common origin for the 5′UTR sites because of their high similarity. The paired miR-1273g-3p and miR-1273d binding sites are located in the 3′UTR at a distance of 13 nucleotides (Figure 9). The homology level of the nucleotide sequences is high not only in the miR-1273g-3p and miR-1273d binding sites but also in the mRNA regions adjacent to these sites.

The paired miR-1273g-3p and miR-1273d binding sites in the CDSs of* ADARB1* and* BEND2* mRNA are shown in Figure 9. The distance between these two binding sites is 13 nucleotides. The nucleotide sequences in the mRNA of the miR-1273g-3p and miR-1273d binding sites are very homologous. Taking into account a deletion of two nucleotides in the 3′-end of the site in* BEND2* mRNA, the homology of the adjacent parts of* ADARB1* and* BEND2* is high (Figure 9). Polypeptides correspond to these sites according to ORFs.

**Figure 10:**
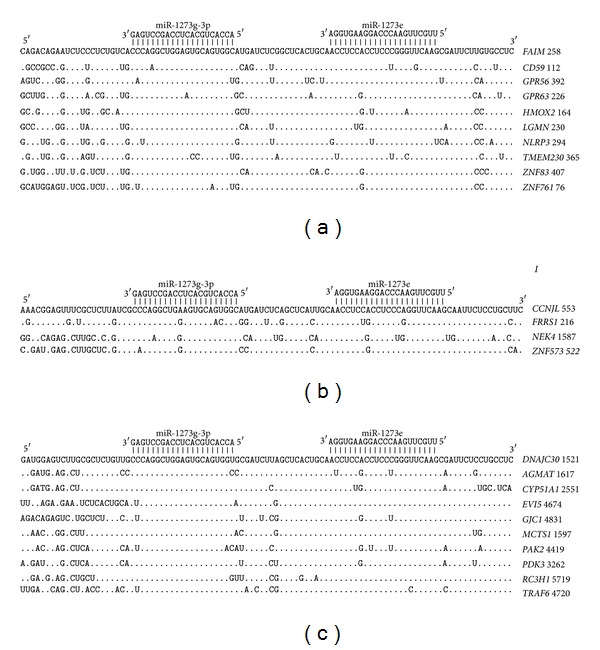
Arranged binding sites miR-1273g-3p and miR-1273e in 5′UTR (a), CDS (b), and 3′UTR (c) mRNA target genes.

The nucleotide sequences of paired miR-1273g-3p and miR-1273e binding sites located in the 5′UTR and their adjacent parts have a high homology level (Figure 10). The mRNA segments in CDSs containing paired miR-1273g-3p and miR-1273e binding sites also have a high degree of homology (Figure 10). The paired miR-1273g-3p and miR-1273e binding sites are found in the 3′UTR of 300 genes, and they have a high degree of homology, as well (Figure 10).

**Figure 11:**
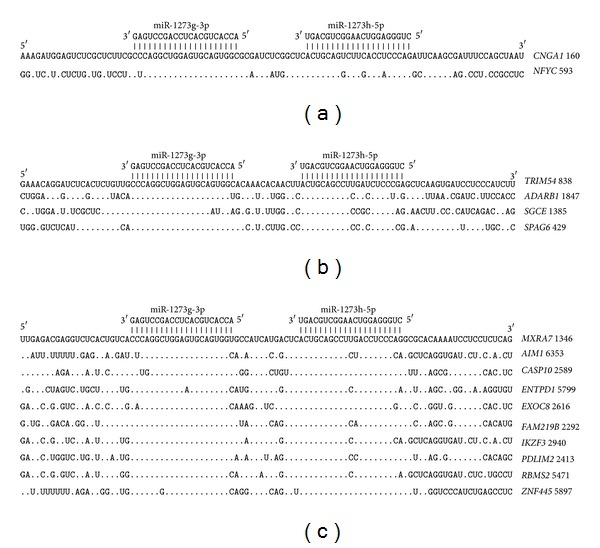
Arranged binding sites miR-1273g-3p and miR-1273h-5p in 5′UTR (a), CDS (b), and 3′UTR (c) mRNA target genes.

The segments of the 5′UTR in the* CNGA1* and* NFYC *genes that contain the miR-1273g-3p and miR-1273h-5p binding sites are shown in Figure 11. All of the nucleotides of these miRNAs form hydrogen bonds in the binding sites, and the degree of their homology is high. The distance between the miR-1273g-3p and miR-1273h-5p binding sites is 12 nucleotides.

The distance between the miR-1273g-3p and miR-1273h-5p binding sites located in the CDSs of four genes is 12 nucleotides. The nucleotide sequences of these binding sites and some adjacent segments have a high degree of homology. The nucleotides of the miR-1273g-3p and miR-1273h-5p binding sites code polypeptides of different ORFs. The LRLECSG and HCNLHL polypeptides are homologous in proteins SPAG6 and TRIM54, while the QAGVQW and LQPPSP polypeptides are homologous in proteins ADARB1 and SGCE (Figure 11). The nucleotide sequences of the 3′UTRs indicate that paired miR-1273g-3p and miR-1273h-5p binding sites are located similarly to those in 5′UTRs and CDSs, with a separation distance of 12 nucleotides (Figure 11). This part of the binding site mRNA is highly conserved, and the adjacent mRNAs are similarly homologous. No paired binding sites are found for miR-1273g-3p and miR-1273h-3p in any of the mRNA locations described above.

### 3.3. Arrangement of the Binding Sites of the miR-1273 Family in mRNA

**Figure 12:**
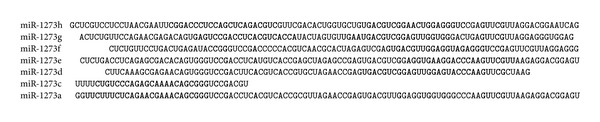
A scheme showing the homology of the pre-miR-1273 family.

This analysis of the localization of paired miR-1273 binding sites in the mRNA of target genes leads to the conclusion that they evolved from a common ancestor. Most of these binding sites are located in mRNA segments 99 nucleotides long (Figures 1–11). Such compactness in the binding site location of the miR-1273 family could be a result of embedding one general nucleotide sequence into the target genes. This work showed that pair binding sites have a monophyletic origin. The complementary nucleotide sequence to pre-miR-1273h includes binding sites for the miR-1273 family, and it is the most probable precursor for these segments (Figure 12). The adaptation of miRNA binding sites to each member of the miR-1273 family or to their combinations could also be due to the evolution of target gene mRNA and their varying functions.

The nucleotide sequences of miR-1273g-3p and miR-1273a have three overlapped nucleotides, as well as pair binding sites (Figure 12). Both miR-1273g-3p and miR-1273c have two overlapped nucleotides and pair binding sites, whose schemes are shown in Figure 8. The nucleotide sequences of miR-1273g-3p and miR-1273h have 16 overlapped nucleotides (Figure 12) that correspond to overlapping of their binding sites, shown in Figure 11. The distance between miR-1273g-3p and miR-1273g-5p is nine nucleotides (Figure 12), which correspond to the interval between the miR-1273g-3p and miR-1273g-5p binding sites, per their schemes (Figure 6). The distances between miR-1273g-3p and miR-1273h-5p (Figure 12) and between their pair binding sites (Figure 11) are each 13 nucleotides. The distance between the nucleotides of miR-1273g-3p and miR-1273d is 13 nucleotides, matching the distances between the pair binding sites of these miRNAs in the schemes of Figure 9. The interval between miR-1273g-3p and miR-1273e is 22 nucleotides (Figure 10), again matching the distance between their pair sites, shown in Figure 12. However, the distance between miR-1273g-3p and miR-1273f is 18 nucleotides (Figure 12) while the distance between their pair sites is only 12 nucleotides (Figures 3 and 4). It is possible that the deletion of six nucleotides occurred in the primary site at an early stage of this pair's formation.

The distances described above between the pair binding sites of the miR-1273 family are nearly always matched in the target gene mRNA. However, all of the pair binding sites of the miR-1273 family have deviations of one-two nucleotides between them. Thus, the average distance between the miR-1273g-3p and miR-1273f mRNA binding sites is 12.1 ± 2.2 nucleotides.

A feature of the miR-1273 family that this study discovered is the presence of pair binding sites in mRNA segments of 100 nucleotides. Figure 12 shows that the miRNA binding sites locate in the mRNAs of target genes occur in a certain order, using different combinations of miR-1273g-3p binding sites and those of other members of this miRNA family.

Increases or decreases in miRNA synthesis, particularly umiRNAs, can lead to an imbalance of gene expressions across the genome. Thus, changes to miRNA expression can lead to disturbances in metabolic processes, the achievement of an organism's development program, an organism's response to different impacts, or ultimately the development of various pathologies. The role of umiRNAs and other miRNAs is assumed to be vast because they circulate in the blood, and almost all of the cells in an organism are available to them [20, 21].

Highly conserved binding sites of miR-1273 family in a large number of genes testify about their emergence in the early stages in human evolution. Arranged localization of these binding sites suggests an interconnected development of evolution of miRNAs and their target genes.
